# Derivation and Osmotolerance Characterization of Three Immortalized Tilapia (*Oreochromis mossambicus*) Cell Lines

**DOI:** 10.1371/journal.pone.0095919

**Published:** 2014-05-05

**Authors:** Alison M. Gardell, Qin Qin, Robert H. Rice, Johnathan Li, Dietmar Kültz

**Affiliations:** 1 Department of Animal Science, University of California Davis, Davis, California, United States of America; 2 Department of Environmental Toxicology, University of California Davis, Davis, California, United States of America; Georgetown University, United States of America

## Abstract

Fish cell cultures are becoming more widely used models for investigating molecular mechanisms of physiological response to environmental challenge. In this study, we derived two immortalized Mozambique tilapia (*Oreochromis mossambicus*) cell lines from brain (OmB) and lip epithelium (OmL), and compared them to a previously immortalized bulbus arteriosus (TmB) cell line. The OmB and OmL cell lines were generated without or with Rho-associated kinase (ROCK) inhibitor/3T3 feeder layer supplementation. Although both approaches were successful, ROCK inhibitor/feeder layer supplementation was found to offer the advantages of selecting for epithelial-like cell type and decreasing time to immortalization. After immortalization (≥ passage 5), we characterized the proteomes of the newly derived cell lines (OmB and OmL) using LCMS and identified several unique cell markers for each line. Subsequently, osmotolerance for each of the three cell lines following acute exposure to elevated sodium chloride was evaluated. The acute maximum osmotolerance of these tilapia cell lines (>700 mOsm/kg) was markedly higher than that of any other known vertebrate cell line, but was significantly higher in the epithelial-like OmL cell line. To validate the physiological relevance of these tilapia cell lines, we quantified the effects of acute hyperosmotic challenge (450 mOsm/kg and 700 mOsm/kg) on the transcriptional regulation of two enzymes involved in biosynthesis of the compatible organic osmolyte, *myo*-inositol. Both enzymes were found to be robustly upregulated in all three tilapia cell lines. Therefore, the newly established tilapia cells lines represent valuable tools for studying molecular mechanisms involved in the osmotic stress response of euryhaline fish.

## Introduction

Fish cell culture has recently offered a number of unique contributions to environmental physiology and toxicology [1], [2]. Cultured cells and tissues offer the advantage of not requiring the sacrifice of live animals and can also provide easier linkage between cause and effect due to controlled manipulation of experimental variables. Originally, fish cell lines were almost exclusively used for the isolation of viruses and diseases, but recently there has been rapid expansion in the derivation and application of these *in culture* systems [3]. For example, fish cell lines have been successfully used for studying toxicology, immunology, carcinogenesis, genetic regulation and expression, and DNA replication and repair [3], [4]. Cell lines from aquaculture species (e.g., salmon, trout, carp) represent a large portion of fish cell lines available today [3], [4]. Such cell lines offer researchers a convenient system to isolate and test the effects of pathogens (e.g., viral susceptibility) and other environmental parameters that are relevant to aquaculture production of these species.

Immortalization of cells is the process by which primary cells are altered such that they can divide continuously for a theoretically infinite number of generations [5]. Cellular senescence is the main hurdle to overcome when establishing new cell lines for long-term culture [6]. All somatic cells typically experience a gradual decrease in proliferative rate and eventually display cellular senescence after a finite number of divisions, which is induced by one or more of the following: telomere erosion, oncogene inhibition, or stress [7]. Timing of senescence can be highly variable among cell types and depends on the species, tissue of origin, and age of the donor organism. Currently, the main methods of immortalization are via the introduction of viral oncogenes (e.g., SV40, adenoviruses) and overexpression of telomerase catalytic subunits (hTERT) [5], [8]. Spontaneous immortalization is also reported to result in the generation of cell lines, especially when cultures are kept for long periods of time at high densities [5].

Recent studies demonstrated that human epithelial cells can be effectively immortalized using a combination of Rho-associated kinase (ROCK) inhibitor and mouse 3T3 irradiated feeder layer [9], [10]. ROCK is an effector protein found downstream of the Rho A pathway, which is involved in actin cytoskeletal organization [11]. Through inhibition of ROCK, epithelial cells are able to bypass senescence and effectively immortalize. These cells are termed “conditionally reprogrammed cells” because they are karyotype stable and non-tumorigenic [10], [12]. ROCK inhibitor has also been reported to be effective in increasing human stem cell survival [13] and suppresses apoptosis in mouse epithelial cells [14], mouse embryonic stem cells [15], and mouse intestinal epithelial stem cells [16]. Major advantages of this technique include that cells retain much of their original phenotype (e.g., cell type-specific differentiation pattern) and faster time to immortalization, which may allow for greater consistency between *in vivo* and *in culture* studies. This is an especially appealing quality for studies investigating environmental toxicology and physiology where physiological relevance is highly valued.

As of yet, the application of ROCK inhibitor/feeder layer has not been tested in the immortalization of fish cells. A major goal of this study was to generate immortalized cell lines from Mozambique tilapia (*Oreochromis mossambicus*) that are amenable to study the mechanisms of the osmotic stress response. *O. mossambicus* has emerged as a model fish system for studying the mechanistic basis of the physiological response to salinity challenge, primarily due to its extreme salinity tolerance [17]. Tilapia cell lines have been established by several workers from brain, ovary, and heart tissues [18]–[20], however, the importance of these cell lines in addressing environmentally-relevant questions has not yet been addressed. In particular, the development of epithelial cell lines for tilapia would provide an excellent tool for investigating molecular mechanisms underlying the response to hyperosmotic challenge since such cells are at the interface of potentially variable osmotic environments and responsible for transepithelial ion transport. Recently, immortalized cell lines derived from other fish species have been utilized for studies focused on responses to environmental challenge [21] and investigation of stress response elements, such as heat shock protein expression [22]. These studies and others have shown that the findings of *in culture* studies can be informative and physiologically meaningful.

A number of molecular and physiological mechanisms have been identified as key contributors to the high salinity tolerance observed in tilapia. One example is the *myo*-inositol biosynthesis (MIB) pathway, which is a highly inducible component of the response to hyper-saline conditions in several tissues of tilapia, including gill epithelium [23], [24], and therefore, represents a suitable system for evaluating the physiological capacity of newly derived tilapia cell lines with regard to cellular osmotic stress responses. *Myo*-inositol is an organic compatible osmolyte that is accumulated in cells when exposed to hyperosmotic stress [25], [26]. In tilapia, the two main enzymes of the MIB pathway, *myo*-inositol phosphate synthase (MIPS) and inositol monophosphatase (IMPA), are robustly induced at the mRNA and protein levels following hyperosmotic exposure [24], [27], [28]. Mammalian systems have at least two different splice variants of MIPS derived from the same gene [29] and two or more isoforms of IMPA from at least two different genes [30], [31]. Recent work in tilapia brain has indicated that MIPS splice variants may be differentially regulated by plasma osmolality [27]. Several studies have also demonstrated that *myo*-inositol concentration increases in multiple tissues of tilapia following acclimation to hyperosmotic conditions [27], [28], [32].

In this study, our goal was to derive several immortalized tilapia cell lines and characterize their osmotolerance and physiological relevance with regard to cellular osmotic stress responses. The MIB pathway was selected for validating the use of cell lines for future studies that investigate response mechanisms to osmotic challenges because it is highly robust in the *in vivo* model and is also very specific to osmotic stress.

## Methods

### Animals

#### Ethics Statement

All animal procedures used in this study were approved by the UC Davis Institutional Animal Care and Use Committee protocol number 13468.

Adult Mozambique tilapia (*O. mossambicus*) were kept in de-chlorinated freshwater at 25–27°C in 208 L recirculating tanks at the UC Davis Cole B Animal Facility. The room was kept on a 12 hr∶12 hr (light:dark) photoperiod, and fish were fed daily with a commercial trout pellet diet (Nelson's Sterling Silver Cup, Murray, Utah, USA) at approximately 1% of fish body mass.

### Derivation of Tilapia Cell Lines

#### Brain Cell Line (OmB) – Spontaneous Immortalization

The primary explant method [5] was used to derive a spontaneously immortalized cell line from tilapia. A male adult fish was first collected and over-anesthetized with tricaine methanesulfonate (MS-222, Argent Chemical Laboratories, Redmond, WA, USA) followed by spinal transection. Brain tissue was removed from the head region after decapitation and immediately incubated in a cocktail of antibiotics (50 µg/µl gentamicin, 250 ng/µl amphotericin B) prepared in Leibovitz L-15 medium (Mediatech, Manassas, VA, USA) containing 10% fetal bovine serum, 100 U/ml penicillin, 100 µg/ml streptomycin for 20–30 min. Following the incubation period, a series of three washes in 1X phosphate buffered saline solution (PBS, Life Technologies, Grand Island, NY, USA) containing the above listed antibiotics was performed while cutting the brain tissue into very small (1–2 mm) pieces using sterilized forceps and scissors. Tissue pieces were then blotted on a kim wipe and seeded in several vented T-25 flasks (BD Biosciences, San Jose, CA, USA). Tissue pieces were left to set on plastic flasks before adding medium for 10–15 minutes to allow for better adherence to the plastic substrate. L-15 medium containing 10% fetal bovine serum, 100 U/ml penicillin, and 100 µg/ml streptomycin was used to carefully submerge the tissue pieces and culture dishes were incubated at 28°C. The tissue explants were then subcultured by trypsinization (0.25% trypsin-EDTA) into new flasks in the same medium and incubated with no supplemental CO_2_ using a 1∶2 splitting ratio for several months until a continuous line was derived. Cells were passaged when flasks were at least ≥80% confluent, which generally took 2–4 weeks to reach.

#### Lip Cell Line (OmL) – ROCK inhibitor/3T3 feeder layer supplementation

DMEM/F12 (2∶1) medium supplemented with fetal bovine serum (5%), hydrocortisone (0.4 µg/ml), adenine (0.18 mM), insulin (5 µg/ml), transferrin (5 µg/ml), triiodothyronine (20 pM), epidermal growth factor (10 ng/ml) (Biomedical Technologies, Inc., Stoughton, MA, USA) was used for lip cultures following the initial explant set-up described above using L-15 medium. 10 µM of ROCK inhibitor compound (Y-27632, Chemdea, Ridgewood, NJ, USA) was also directly added to DMEM/F12 complete medium following the first day. Additionally, lethally irradiated 3T3 cells [33] were co-cultured with the lip cells to allow for the proliferation of targeted epithelial cells [34]. OmL cells were incubated at 28°C with 5% CO_2_ supplementation.

#### Bulbus arteriosus Cell Line (TmB

The immortalized TmB cell line [20] was generously provided by Dr. Ronald Hedrick and Susan Yun from the UC Davis Veterinary College (Table 1). TmB cells were maintained in L-15 medium containing 10% FBS, 100 U/ml penicillin, and 100 µg/ml streptomycin and were incubated at 28°C with no supplemental CO_2_. Cells were passaged every 3–4 days using a 1∶3 splitting ratio.

**Table 1:** Properties of all tilapia cell lines used in this study, including a previously derived line, TmB, and two newly derived lines, OmB and OmL.

Cell Line	Immortalization Method	Year Established	Tissue Origin	Phenotype	Period of Crisis	Time to Immortalization	Passage # at Immortalization	Reference
TmB	Not reported	1985	Bulbus arteriosus	Endothelial	Not reported	Not reported	Not reported	Lewis and Marks, 1985
OmB	Spontaneous	2010	Brain	Fibroblast-like	∼3–4 months	∼8 months	8	This study
OmL	Y-27632+3T3 Feeder Layer	2012	Lip	Epithelial-like	None	∼4 months	5	This study

### Fluorescent Staining

Cells of each line (OmB: Passage 17, OmL: Passage 16, TmB: Passage 71) were seeded on plastic coverslips (Sarstedt, Newton, NC, USA) that were held in 6-well plates (Corning, Tewksbury, MA, USA) with complete L-15 medium. After two days, coverslips were incubated with Mitotracker Red and Hoechst 33342 live stains (Life Technologies, Grand Island, NY, USA) to label mitochondria and nuclei, respectively, and cells were visualized using a fluorescent microscope (Olympus, Center Valley, PA, USA).

### Osmotolerance Evaluation

#### Exposure Conditions

Cells of each line (OmB: passage 10, OmL: passage 7, TmB: passage 63) were initially seeded on 12-well plates (Corning, Tewksbury, MA, USA or BD Biosciences, San Jose, CA, USA) and grown to approximately 90% confluency. The day before experiments, cell medium was aspirated and replaced with fresh complete L-15 media. Cells were then exposed to a range of osmolalities in 12-well plates (Corning, Tewksbury, MA, USA or BD Biosciences, San Jose, CA, USA) from baseline medium (315±5 mOsm/kg) to 900 mOsm/kg using 50 mOsm/kg increments by supplementing medium with sodium chloride (Sigma-Aldrich, St. Louis, MO, USA, Cell culture grade). One-fifth the total medium volume per well was replaced with a hyperosmotic stock solution to obtain the targeted final osmolality value. Targeted final osmolality was confirmed by taking a small aliquot of medium and measuring the osmolality using an osmometer (Advanced Instruments, Inc., Norwood, MA, USA).

#### Cell Morphology

Cells were exposed to the different osmolality media and were visually inspected for changes in morphology every 8 hours using an inverted microscope (VWR International, Radnor, PA, USA). Percent confluency was also determined at each 8 hour increment within a 24 hour period and was documented with digital images.

#### Cell Viability Assay

A cell viability assay was coupled with visual inspection of osmotolerance (see above) to obtain a quantitative measure for cell survival. At 24 hours, cells grown in a 12-well plates (Corning, Tewksbury, MA, USA or BD Biosciences, San Jose, CA, USA) were washed with 1X PBS, trypsinized, and the cell suspension was diluted (1∶9) in a 0.2 µm filtered electrolytic solution (Beckman Coulter, Indianapolis, IN, USA). A blank with Coulter salt solution and medium was used and subtracted to determine the final cell number. Cells plots were obtained for each replicate (n = 3) and cell density in the 10–40 µm size range was quantified. The number of live cells per replicate was quantified using Multisizer3 Coulter Counter software (Beckman Coulter, Indianapolis, IN, USA) and values were averaged for each treatment osmolality and used to generate an osmotolerance curve. Maximum acute osmotolerance (OT_max_) was defined as the concentration at which approximately 50% of the number of live cells remained attached to the substrate of the plastic dish following acute hyperosmotic challenge for 24 hours. The OT_max_ was extrapolated from the osmotolerance curve for each cell line.

### Hyperosmotic MIB Pathway Activation

Plasma osmolality values from *in vivo* treatment of tilapia [27] were used to determine a physiologically relevant target osmolality value of 450 mOsm/kg for the acute *in culture* exposures of cell lines. The combined OT_max_ value used was 700 mOsm/kg and was determined as described above.

#### Acute Exposure

OmB (passage 11), OmL (passage 10), and TmB (passage 63) cells were seeded in 6-well plates (Corning, Tewksbury, MA, USA for OmL or BD Biosciences, San Jose, CA, USA for OmB and OmL) and allowed to grow to 80–90% confluency. Hyperosmotic stock solutions were made by supplementing L-15 medium with sodium chloride (Sigma-Aldrich, St. Louis, MO, USA) and then confirming the osmolality with an osmometer (Advanced Instruments, Inc., Norwood, MA, USA). One-fifth of the total medium in wells (n = 4) was replaced with a hyperosmotic stock solution to reach the target osmolalities of 450 mOsm/kg (physiologically relevant) and 700 mOsm/kg (maximum acute osmotolerance). Cells were harvested by adding an extraction buffer (Qiagen, Hilden, Germany) supplemented with β-mercaptoethanol to wells of the plate combined with scraping at 4, 8, 16, and 24 hours.

#### Gradual Exposure

OmB cells (passage 11) were seeded in 6-well plates and allowed to grow to ∼70% confluency. Osmolality was increased in increments of 75 mOsm/kg every 24 hours by replacing one-fifth of the medium in wells (n = 4) until the final osmolality (450, 600, and 750 mOsm/kg) was reached. Cells were then held at the targeted endpoint osmolality for 24 hours after the final osmolality increase and before collection. All cells were harvested using a 10X Tryple solution (Life Technologies, Grand Island, NY, USA) at the end of the exposure period and cell pellets were stored at −80°C until analysis.

#### Controls

Cells of a tilapia cell line underwent identical volume replacement with iso-osmotic medium (315±5 mOsm/kg) in wells (n = 4) with complete medium to account for handling stress induced in acute and chronic acclimations. Cells were harvested in the wells at 4, 8, 16, and 24 hours for RNA using a lysis extraction buffer (Qiagen, Hilden, Germany).

### mRNA Expression Assay

#### Reaction conditions

Quantitative real-time PCR (qRT-PCR) was performed to measure mRNA abundance levels of MIPS splice variants (MIPS-160, MIPS-250) and IMPA isoforms (IMPA1, IMPA2) as described previously [23], [24], [27]. Briefly, total RNA was extracted from tilapia cell line extracts from both acute and chronic acclimations using the RNeasy Kit (Qiagen, Hilden, Germany) with a direct lysis procedure in 6-well plates. RNA was treated with Turbo-DNAfree (Promega, Madison, WI, USA) in order to remove any DNA contamination in the samples. Complementary DNA (cDNA) was then synthesized by reverse transcribing total RNA using Superscript III (Life Technologies, Grand Island, NY, USA) with a 1∶1 mix of random hexamers and oligo(dT) as primers. PCR products of targeted genes (Table 2) were visualized using cDNA as template on a 2% agarose gel to confirm expected product size. Prior to quantification, a standard curve was run to determine dilutions of cDNA for each primer set. Samples were run in duplicate on a 96-well plate using Sybr Green as the method of detection. β-actin and 18S rRNA, which do not change in response to hyperosmolality in tilapia tissues [23], [24], [27], were used as reference genes.

**Table 2:** Primers and cDNA dilutions used for qRT-PCR.

Gene	Forward (5′ to 3′)	Reverse (5′ to 3′)	Product Size (bp)	Accession Number	cDNA Dilution
MIPS-160	CAGAGTCGCGCAGACAATGT	CGTTGACCCCTGGGATGATA	164 and 251	DQ465381	1∶9
MIPS-250	GTGCATGATCTTCCAGATGGAGCG	AGAAGCGCTCGGTGTTGGCG	110	Sacchi et al., 2013	1∶9
IMPA1	CGAAACTCTCCTAAGCAAGCCCCC	CCAGCTTTCCTAATTTCCGCGCCA	114	JQ943581	1∶9
IMPA2	TACCAGAATCCTCTTCTTGGCCACACC	ACCAGGACACTGATGCACAGCTA	121	XM_003439148	1∶9
β-actin	CCACAGCCGAGAGGGAAAT	CCCATCTCCTGCTCGAAGT	104	AB037865	1∶9
18S rRNA	CGATGCTCTTAGCTGAGTGT	ACGACGGTATCTGATCGTCT	260	AF497908	1∶729

#### qRT-PCR Data Analysis

Target C_t_ values for MIB enzymes were first normalized against the reference genes (β-actin and 18S RNA) after correcting by efficiency using LinRegPCR software [35]. C_t_ values were then converted to a fold ratio value using the Pfaffl method, which accounts for differences in primer pair efficiency [36]. Data presented are normalized against β-actin, but using 18S rRNA produced similar results (data not shown).

### Proteomics

OmB (passage 19) and OmL (passage 9) cells were grown in iso-osmotic conditions as described above on 35 mm dishes and harvested when they approached confluency. The proteomes of four technical replicates for each of the two cell lines were analyzed by online LCMS. Proteins were extracted, digested with immobilized trypsin, and buffer exchanged to resuspend tryptic peptides in 3% acetonitrile/97% LCMS-grade water as previously described [37]. Peptides were injected in 2 µl volume corresponding to 330 ng total peptide amount using a nanoAcquity sample manager (Waters) and separated after 3 min sample trapping (Symmetry, Waters 186003514) on a 1.7 µm particle size BEH C18 column (250 mm×75 µm, Waters 186003545) by reversed phase chromatography using a nanoAcquity binary solvent manager (Waters). A 130 min linear solvent gradient ranging from 3% to 35% acetonitrile (ACN) was employed, internal calibration performed, and mass spectra acquired as previously described [37] except that an ImpactHD mass spectrometer (Bruker Daltonics) was used. Raw data were converted into mzXML format using DataAnalysis 4.2 (Bruker Daltonics) and PEAKS suite 7 (BSI, Inc.), Mascot 2.2.7 (Matrixscience, Ltd.), and X!Tandem Cyclone (The GPM) were used for protein identification using the following parameters: enzyme specificity  =  trypsin, max. missed cleavages permitted  = 2, fixed modification  =  Cys carbamidomethylation, variable modifications  =  Met oxidation, Pro hydroxylation, N-terminal acetylation, Pyro-Glu at N-terminal Gln and Gln/Asn deamidation, precursor ion mass tolerance  = 15 ppm, fragment ion mass tolerance  = 0.02 Da. A threshold score of 5% probability that a protein identification is incorrect was used for accepting individual MS/MS spectra. A RefSeq database containing 28,020 protein sequences, including the complete predicted *Oreochromis niloticus* proteome and all available *Oreochromis mossambicus* sequences, was downloaded from NCBI on March 01, 2014. PEAKS suite 7 was used to double the size of this database by adding a randomly scrambled decoy entry for every sequence and enabled determination of protein false discovery rate (FDR). An FDR of 1% was considered the upper limit for protein identification. Quantitative label-free comparison of OmL and OmB proteomes was performed using PEAKS suite 7 according to manufacturer instructions based on extracted ion chromatogram peak areas of detectable isotopes for unique peptides that identify the corresponding protein (PEAKS 7 Quant module, BSI, Inc.). Total ion chromatograms of the corresponding LCMS dataset were used for normalization.

### Statistical Analysis

Analysis of variance (ANOVA) was performed separately on data obtained from each cell line using IMB SPSS software (v. 21). Normality and homoscedasticity of variance were assessed prior to ANOVA using Wilk-Shapiro and Levene tests, respectively. On occasion, a statistically significant outlier was removed to meet the assumption of normality if this could not be achieved through transformation of data. To determine if there was an effect of sampling time on control cells, a one-way ANOVA with time as a factor was performed for all iso-osmotic (315±5 mOsm/kg) samples at every time point or osmolality endpoint. If non-significance was determined (data not shown), all control samples were grouped together in a zero time point. Since variance heteroscedasticity was generally observed in data sets, weighted least squares (WLS) were conducted by grouping samples by the factor of time [38]–[40]. A weight for each sample grouping was calculated by taking the inverse of the unstandardized residual variance per assigned group. Subsequently, a one-way WLS ANOVA was performed with time or salinity as a factor which compared salinity-treated groups to the combined iso-osmotic control group (0 hr). Post-hoc analyses were then conducted using Tukey's multiple comparison test or Dunnett's test (comparisons with control only) to determine significant differences between time points or salinity endpoints for each cell line. Significance was set at p<0.05 for statistical tests. For proteomics data, statistical analyses were performed using a standardized software package (PEAKS suite 7, BSI, Inc.) in order to compare protein abundances in OmL and OmB cell lines. Significance was reported as -10logP values (PEAKS suite 7, BSI, Inc.).

## Results

### Cell Line Derivations

**Figure 1:**
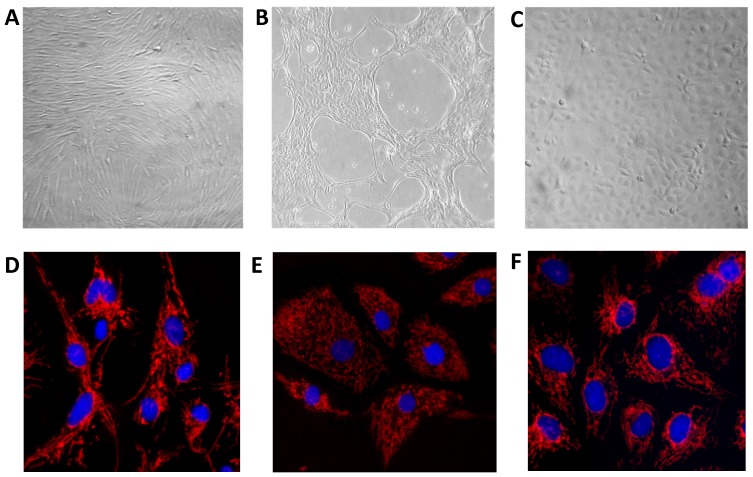
Cell morphology of tilapia cell lines. Images depicting differences in cell morphology of two newly immortalized cell lines derived from brain (OmB) (A+D) and lip (OmL) (B+E) and the previously established bulbus arteriosus (TmB) (C+F) cell line. OmB cells are fibroblast-like, OmL cells are epithelial-like, and TmB cells are endothelial. Images were taken on an inverted or fluorescent microscope at 100X (A–B) and 250X (C), and 400X (D–F) magnification. Cells (D–F) were stained with MitoTracker Red to visualize mitochondria (red) and with Hoechst 33342 for nuclei (blue).

The OmB cell line was judged to be immortalized after approximately six months of continuous culture. Cells from the original tissue explants divided much faster (1–3 days to confluency) than those in isolated colonies (weeks-months to confluency). Early passaging was generally more successful if tissue pieces from the initial primary culture system were carried over with the trypsinized cells. Alternatively, explants from the initial primary culture could also be regenerated from the original tissue piece by adding medium to the culture dish after trypsinized cells were seeded into new dishes. Small colonies from these trypsinized cells were often visible after early passaging; however, it took several weeks to months before the colonies reached confluency and were ready to be passaged again. Changes in cell morphology were also observed in OmB as cells were continually passaged within the first few months. In the initial stages, cultures appeared to have multiple cell morphologies. The beginning colonies had a combination of star shaped (astrocyte-like) or long and slender (fibroblast-like) cells, followed by an extended period of cell crisis (∼3–4 months). During the cell crisis event, the majority of cells showed signs of deterioration (e.g., vacuolization) which led to eventual cell death of these previously viable cells. Cells displaying a typical fibroblast-like phenotype were observed approximately four months post crisis stage and eventually dominated the culture as a continuous cell line after approximately eight successful passages. The initial growth of these cells was poor, but the growth properties of OmB continually improved with further subculturing. These fibroblast-like cells displayed a slender, spindle-like morphology and grew in parallel with neighboring cells to produce characteristic large colonies that resembled waves (Figure 1A).

The OmL cell line was successfully immortalized using a ROCK inhibitor with 3T3 feeder layer culture after approximately five successful passages. Effective immortalization of lip epithelial-like cells took approximately four months. Passaging was typically unsuccessful for lip epithelial cells without supplementation with ROCK. Epithelial-like cells were observed in the explant outgrowths, and this morphology was maintained. OmL cells were rounded in shape, but elongated cells were occasionally observed in the culture (especially at sub-confluency, Figure 1B). OmL cells were noted to have a very slow division rate at low passage states, but no period of cell crisis was observed.

The previously derived TmB cell line displayed an endothelial-like morphology that is consistent with an earlier report [20]. Cells were generally rounder in shape compared to OmB and OmL cell lines (Figure 1C). TmB cells did not exhibit phenotypic change when cultured in complete L-15 medium.

Cell morphology was found to be stable in the two newly derived OmB and OmL cell lines following immortalization. The morphology of TmB cells was also stable with subsequent passaging, however the passage number used for TmB experiments was generally much higher than that of OmB or OmL because it was previously derived. Differences in passage number used for the various experiments described above did not result in changes in morphology or responses to exposures.

### Validation of Species Specificity

Tilapia-specific primers were tested against cDNA of the three cell lines and PCR products visualized on an agarose gel (data not shown) to verify species origin (*O. mossambicus*) of each cell line. PCR products were observed for MIPS-160, MIPS-250, IMPA1, IMPA2, β-actin, and 18S rRNA in the expected size ranges.

### Proteomic Comparison of OmB and OmL Cell Lines

**Figure 2:**
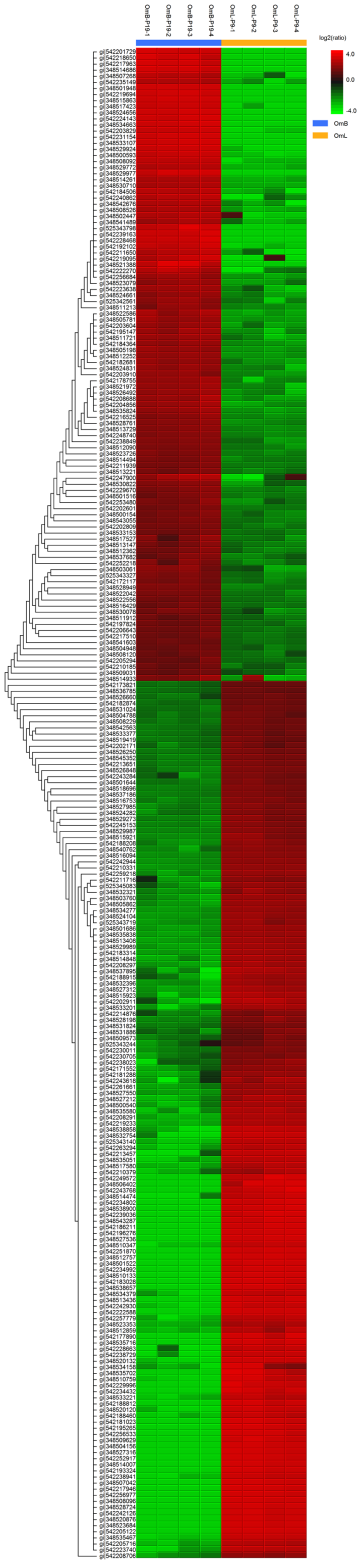
Heat map representing comparative proteomics data for newly derived OmB and OmL cell lines. Data presented are significantly different ratios of protein abundances (relative to OmB abundance) for four technical replicates of OmB and OmL generated with a proteomics software package (PEAKS suite 7, BSI, Inc.). Protein accession numbers and relationships are given on the left side of the heat map.

1612 proteins and 975 unique protein groups were identified for OmB while 1565 proteins and 1013 unique protein groups were identified for OmL (Files S1 and S2). 249 proteins were identified by PEAKS suite 7 software (BSI, Inc.) as being expressed at a level greater than a two-fold change (−10logP significance values reported in Files S3 and S4) when comparing proteomes of OmB and OmL cell lines (Figure 2). In OmB, a number of brain-specific cell markers were observed at higher level than in OmL. These targets included astrocytic phosphoprotein PEA-15-like (348534663), hematological and neurological expressed 1 protein-like (348530822), neurofilament heavy polypeptide-like (348530078), and optineurin-like (542247900) proteins. In contrast, a number of epithelial-like cell markers were highly abundant in the OmL, including keratin type I cytoskeletal 13-like (348510133, 348532754, 542183028, 542242944, 348534379), keratin type II cytoskeletal 8-like (348523353), epidermis-type lipoxygenase 3-like (542238941), tight junction protein ZO-2(542188812), integrin alpha-V-like (542202911), integrin alpha-6-like (542205122), integrin beta-4-like (542214876), cingulin-like protein 1-like (542188915), junction plakoglobin-like (542242930), catenin alpha-1 (348516753), laminin subunit alpha-3 (542205716), laminin subunit gamma-2-like (542257779), and laminin subunit beta-3-like (542259218) proteins.

### Osmotolerance Testing

**Figure 3:**
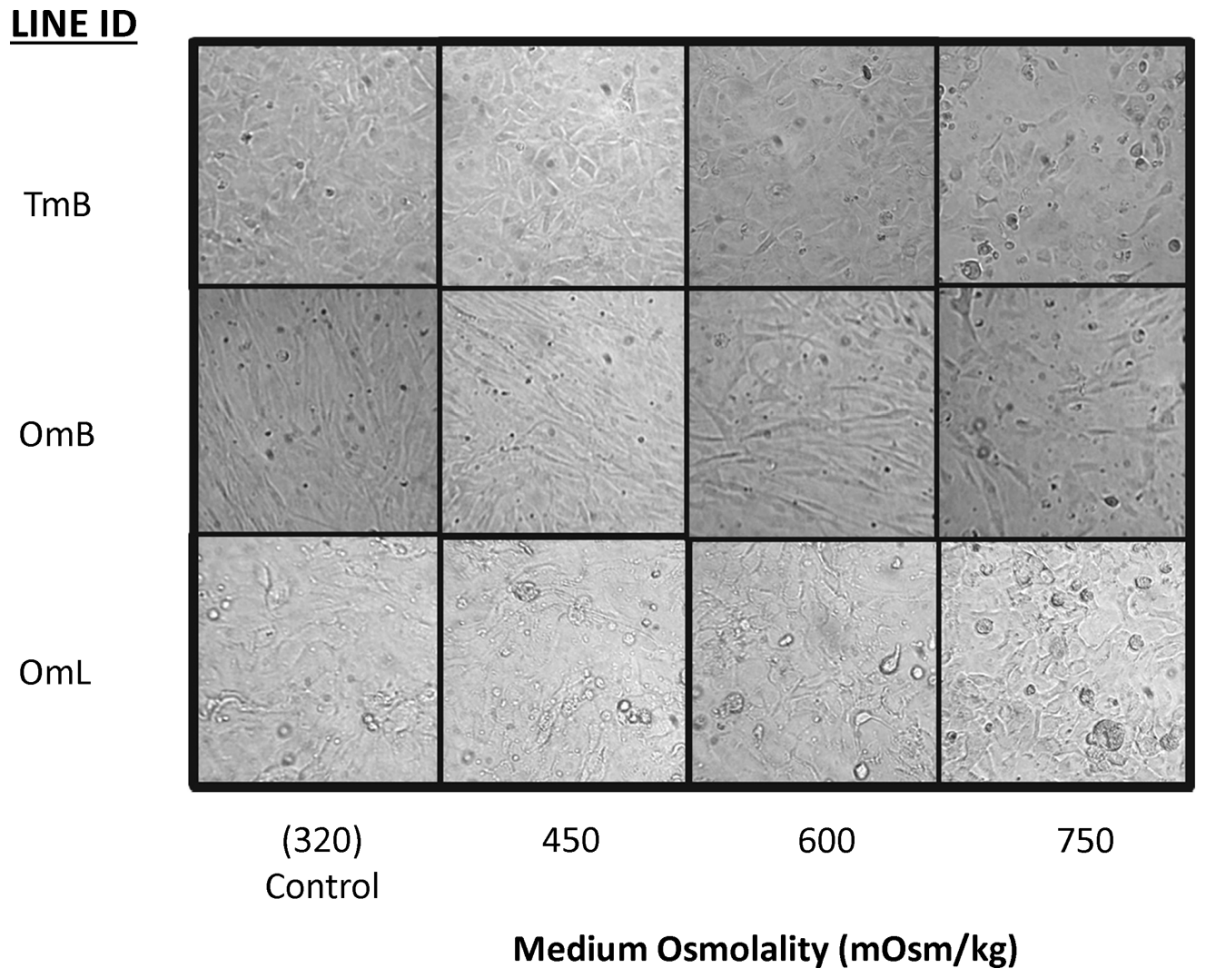
Salinity-induced changes in morphology and confluency. Cell morphology and confluency changes observed after three cell lines (OmB, OmL, TmB) are exposed to acute hyperosmotic stress (450, 600, 750 mOsm/kg) for 24 hours. Images taken on an inverted microscope at 250X magnification.

**Figure 4:**
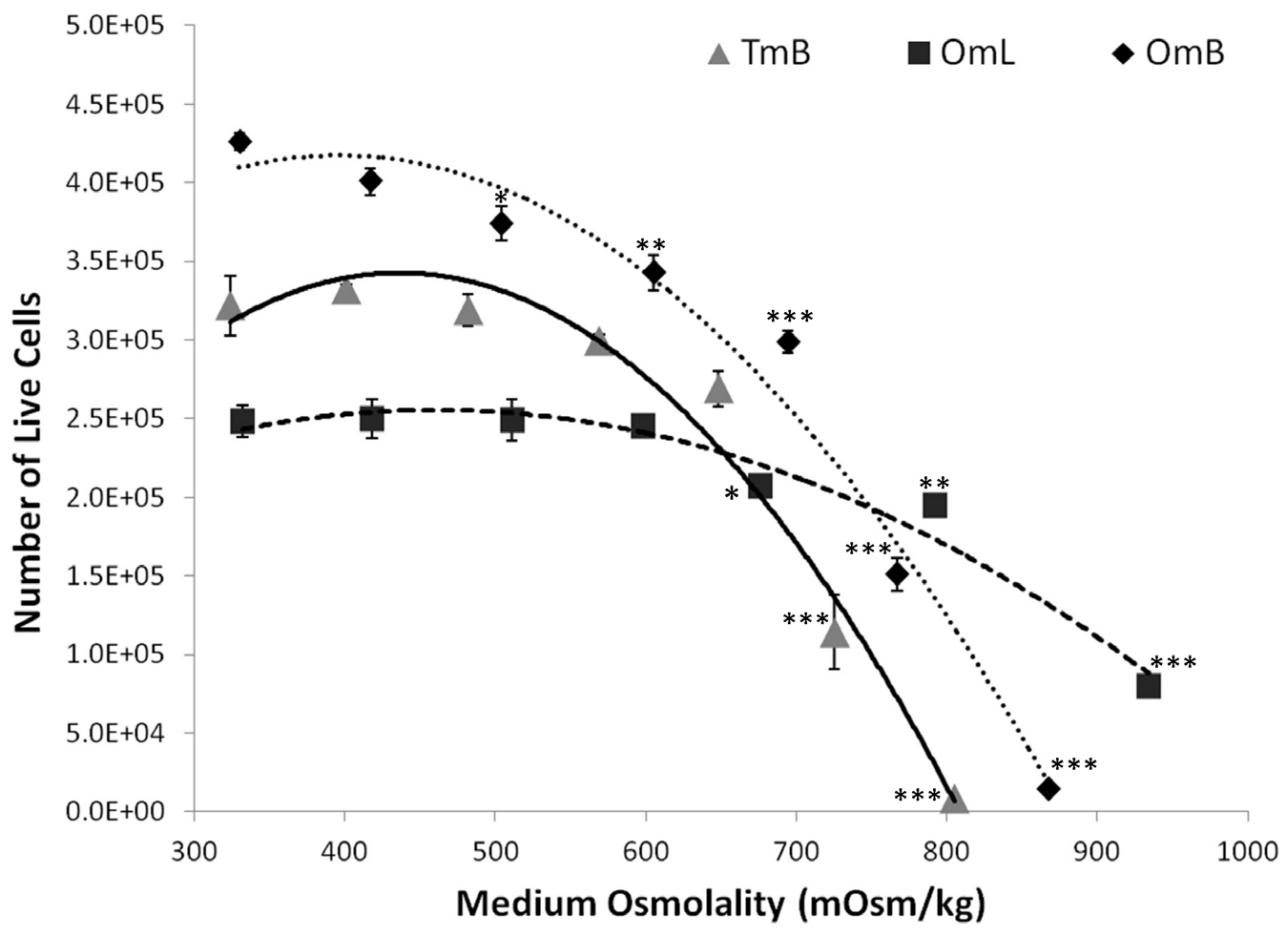
Salinity-induced changes in cell viability. Means ± SEM of number of live cells as a measure of cell viability for three cell lines (OmB, OmL, TmB) exposed to osmolalities ranging from 300–900 mOsm/kg for 24 hours. Cell numbers were measured using the Coulter Counter method. If standard errors are not visible then they are smaller than the size of the symbol for the corresponding data point. *, **, and *** indicates significant differences from control cells at p<0.05, p<0.01, and p<0.001, respectively.

Visual inspection following acute osmotolerance testing indicated that cell attachment and confluency decreased with increasing medium osmolality in all tilapia cell lines (Figure 3). Based on the cell viability assay, OmL cells displayed a shallower slope in the osmotolerance curve compared to OmB and TmB cells (Figure 4), indicating a higher tolerance to sodium chloride. The osmolality which resulted in a 50% reduction of live cells (OT_max_) was not significantly different between OmB (735±2.9 mOsm/kg) and TmB cell lines (706.7±10.9 mOsm/kg). In contrast, the OT_max_ was significantly higher (p<0.001) in OmL (975±16.1 mOsm/kg) than in the other two cell lines. In the OmB cell line, the number of live cells in treated conditions was significantly lower (p<0.05) than in control conditions (iso-osmotic) beginning at 500 mOsm/kg. The number of live cells was significantly lower in TmB (p<0.001) and OmL (p<0.01) cell lines compared to their respective controls starting at 700 mOsm/kg.

### Hyperosmotic MIB Pathway Activation

#### TmB – Acute osmotic stress

**Figure 5:**
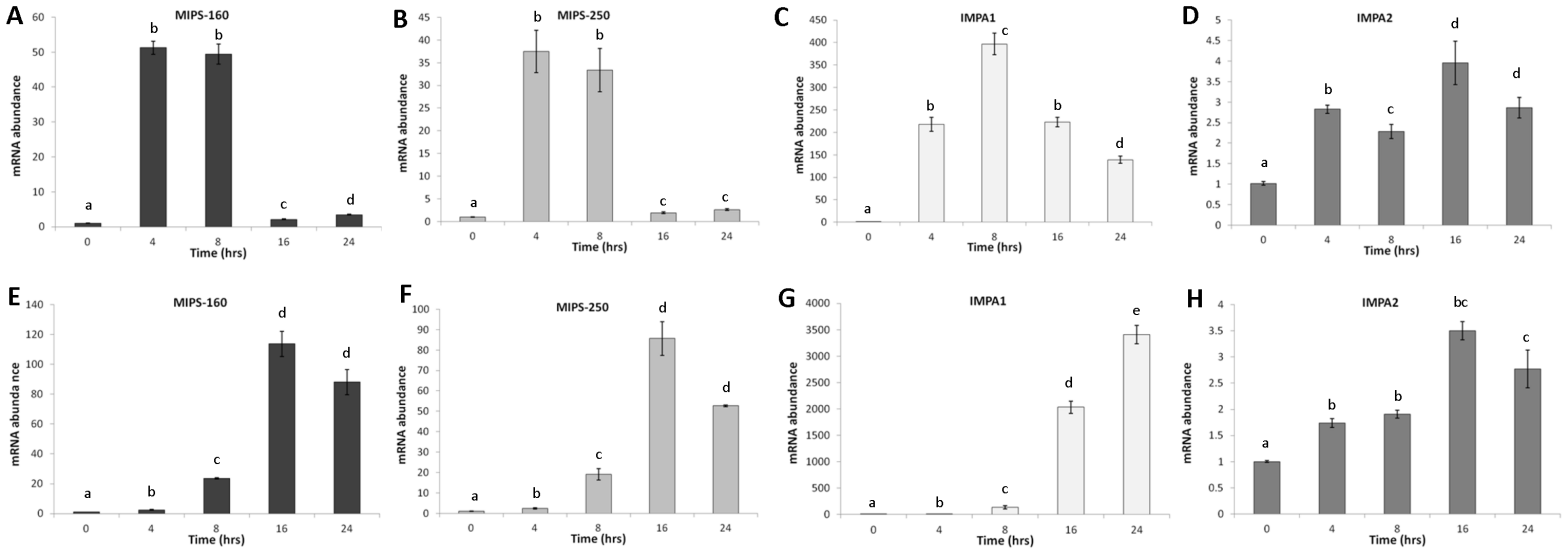
Expression of MIB genes in OmB cell line. Means ± SEM of MIPS-160 (A+E), MIPS-250 (B+F), IMPA1 (C+G), IMPA2 (D+H) mRNA abundance in the TmB cell line derived from bulbus arteriosus endothelium following acute hyperosmotic challenge to 450 mOsm/kg (A–D) and 700 mOsm/kg (E–H) for 4, 8, 16, and 24 hours. Tukey HSD post-hoc comparisons (p<0.05) are indicated with a letter assignments (only within gene comparisons). Groups that share the same letter are not significantly different.

Under 450 mOsm/kg conditions, MIPS-160, MIPS-250, and IMPA1 mRNA expression were significantly upregulated at every time point compared to the control (Figure 5A–B, p<0.001). MIPS-160 and MIPS-250 peaked at earlier time points (4–8 hr) and then quickly returned to lower levels, while IMPA1 peaked at 8 hours (396.9±24.4 fold, p<0.001, Figure 5B). IMPA2 mRNA was also significantly induced at 450 mOsm/kg conditions compared to control cells, but to a much lesser degree than IMPA1. Under 700 mOsm/kg conditions, MIPS-160, MIPS-250, and IMPA1 mRNA abundances were significantly upregulated at every time point compared to control cells. The peak induction of MIPS-160 and MIPS-250 mRNA abundance occurred at 16–24 hours, while the peak induction of IMPA1 mRNA abundance occurred at 24 hours (3407.6±171.1 fold, p<0.001) at 700 mOsm/kg. IMPA2 mRNA abundance was significantly upregulated compared to control cells at 700 mOsm/kg, but the induction level was much lower than for IMPA1.

#### OmB – Acute osmotic stress

**Figure 6:**
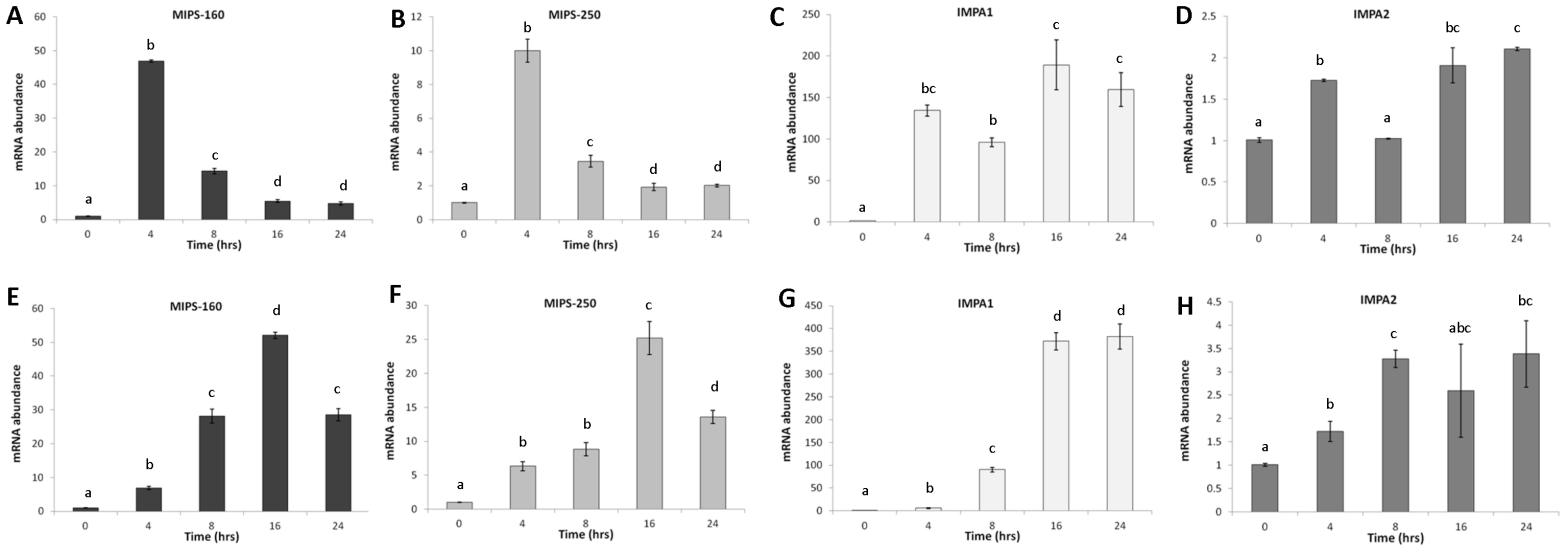
Expression of MIB genes in OmB cell line. Means ± SEM of MIPS-160 (A+E), MIPS-250 (B+F), IMPA1 (C+G), IMPA2 (D+H) mRNA abundance in the OmB cell line derived from brain tissue following acute hyperosmotic challenge to 450 mOsm/kg (A–D) and 700 mOsm/kg (E–H) for 4, 8, 16, and 24 hours. Tukey HSD post-hoc comparisons (p<0.05) are indicated with a letter assignments (only within gene comparisons). Groups that share the same letter are not significantly different.

Under 450 mOsm/kg conditions, MIPS-160, MIPS-250, and IMPA1 mRNA expression was significantly upregulated at every time points compared to the control (Figure 6A–B, p<0.001). MIPS-160 and MIPS-250 mRNA abundances were found to peak at 4 hours and then quickly return to lower levels at later time points. IMPA1 was found to remain elevated (159.4±20.3 fold, p<0.001) after the initial induction at 4 hr under 450 mOsm/kg conditions (Figure 6B). IMPA2 mRNA abundance was also significantly induced at 450 mOsm/kg at 4, 16, and 24 hours compared to control cells, but to a much lesser degree than IMPA1. Under 700 mOsm/kg conditions, MIPS-160, MIPS-250, and IMPA1 mRNA abundances were also significantly upregulated at every time points compared to the control (Figure 6C-D). The peak induction of MIPS-160 and MIPS-250 mRNA abundance occurred at 16 hours while the peak induction of IMPA1 mRNA abundance occurred at 16–24 hours (382.5±27.8 fold, p<0.001) at 700 mOsm/kg. IMPA2 mRNA abundance was also significantly upregulated at 4, 8, and 24 hours compared to control cells under 700 mOsm/kg conditions, but the induction level was much lower than for IMPA1.

#### OmL – Acute osmotic stress

**Figure 7:**
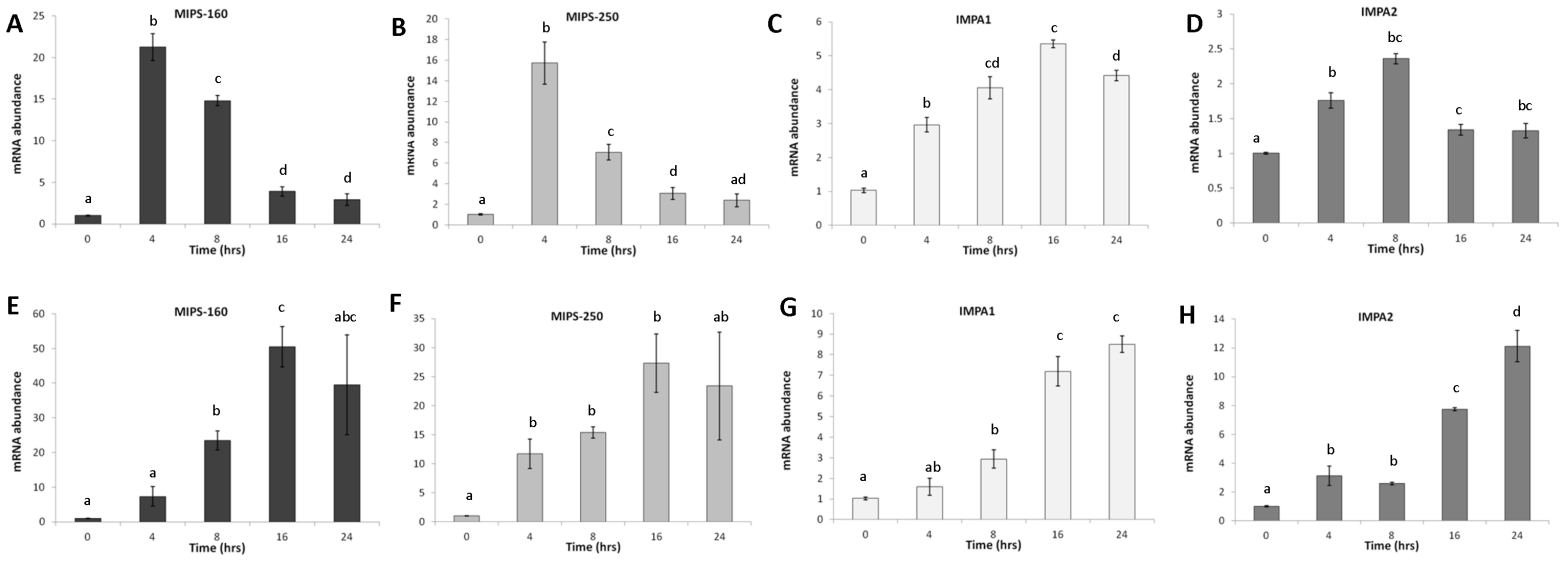
Expression of MIB genes in OmL cell line. Means ± SEM of MIPS-160 (A+E), MIPS-250 (B+F), IMPA1 (C+G), IMPA2 (D+H) mRNA abundance in the OmL cell line derived from lip epithelium following acute hyperosmotic challenge to 450 mOsm/kg (A–D) and 700 mOsm/kg (E–F) for 4, 8, 16, and 24 hours. Tukey HSD post-hoc comparisons (p<0.05) are indicated with a letter assignments (only within gene comparisons). Groups that share the same letter are not significantly different.

At 450 mOsm/kg, MIPS-160, MIPS-250, and IMPA1 mRNA expression was significantly upregulated at every time points compared to the control (Figure 7A–B, p<0.001). MIPS-160 mRNA abundance peaked at 4 hours (21.2±1.6 fold, p<0.001) and then quickly returned to lower levels. Under 450 mOsm/kg conditions, IMPA1 mRNA abundance peaked at 16 hours (5.4±0.1 fold, p<0.001), but remained elevated at later time points after the initial induction (Figure 7B). IMPA2 mRNA was significantly induced at 450 mOsm/kg at every time points compared to control cells and at a similar level as IMPA1. Under 700 mOsm/kg conditions, MIPS-160, MIPS-250, and IMPA1 mRNA abundances were significantly upregulated at most time points compared to the control (Figure 7C–D). Peak induction of MIPS-160 and MIPS-250 mRNA abundance occurred at 16 hours (50.5±5.8 fold and 27.3±5.0 fold, respectively, p<0.001), while the peak induction of IMPA1 mRNA abundance occurred at 16–24 hours (8.5±0.4 fold, p<0.001) at 700 mOsm/kg. IMPA2 mRNA abundance was also found to be significantly upregulated at 700 mOsm/kg at every time point compared to control cells and at a similar or higher level than IMPA1.

#### OmB – Osmolality dependence

**Figure 8:**
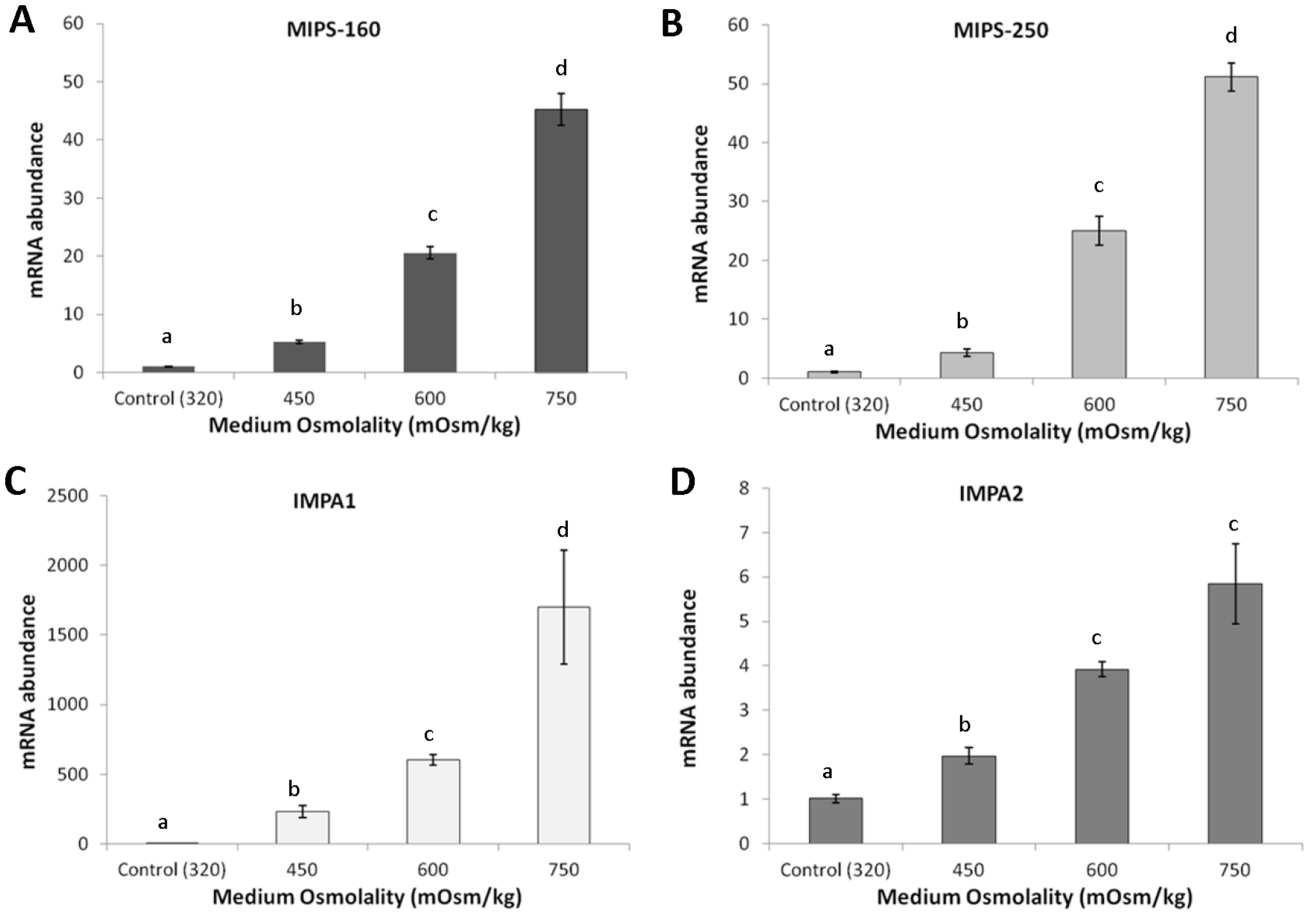
Osmolality dependent MIB gene expression in OmB cell line. Means ± SEM of MIPS-160 (A), MIPS-250 (B), IMPA1 (C), and IMPA2 (D) mRNA abundance in the OmB cell line derived from brain after chronic hyperosmotic challenge (450, 600, 750 mOsm/kg). Tukey HSD post-hoc comparisons (p<0.05) are indicated with a letter assignments (only within gene comparisons). Groups that share the same letter are not significantly different.

To determine whether the degree of MIB pathway *in culture* correlates to medium osmolality, the OmB cell line was used. A classic dose-dependent response was observed with regard to the mRNA expression of MIPS-160, MIPS-250, IMPA1, and IMPA2 with exposure to higher osmolality (Figure 8A–D). MIPS-160 and MIPS-250 mRNA induction levels were similar for a given osmolality (Figure 8A). The level of mRNA induction was highest for IMPA1 at the 750 mOsm/kg exposure level (1697.8±409.6 fold, p<0.001, Figure 8B). IMPA2 was also induced under higher osmolality, but to a much lesser degree than IMPA1 and showed significantly higher abundance levels than control cells at ≥450 mOsm/kg.

## Discussion

### Spontaneous immortalization of tilapia brain cells

Tilapia brain cells were spontaneously immortalized in this study to generate a new cell line without the use of any inducing agent for transformation. Brain-derived primary cultures and cell lines have been established for several other fish species including tilapia [19], another African cichlid [41], goldfish [42], rainbow trout [43]–[45], barramundi [46], sea bass [47] and pompano [48]. We observed that a fibroblast-like phenotype prevailed after primary cells were kept in culture conditions for extended periods of time and overcame crisis. Wen et al. [19] also described that fibroblast-like cells were the most common cell in primary cultures of tilapia brain and were likely radial glial cells or tanycytes, based on positive labeling for glial fibrillary acidic protein and vimentin. A transition of cell types in culture was also noted in a silver sea bream fin-derived fibroblast cell line [49] and a grouper fin cell line [50]. The authors reported that the fibroblasts were evident in the later stages of culture and eventually developed into an continuous line [49]. Fibroblast-like cells have also been observed in mammalian primary epithelial cultures, but can be removed using EDTA [34] or selective media [5]. Spontaneous immortalization of fibroblasts from mammalian cells has also been observed in mammalian cell cultures. In particular, rodent fibroblasts have developed into continuous lines [51]. After crisis, rare genetic variants arise that become continuous lines [52]. These alterations are normally related to the loss of functional rentinoblastoma or p53 proteins, which control two major cell cycle checkpoints [4].

It has not yet been investigated whether the derivation of tilapia cell lines by spontaneous immortalization can also be accomplished from other tissues and whether this method of immortalization always leads to a fibroblast-like phenotype. It is interesting to note that serum factors may inhibit proliferation of some cell types (e.g., epithelial cells), which may explain the eventual domination by fibroblasts [5]. Differences in telomere erosion between species may also contribute to altered rates of success for spontaneous immortalization in subsequent cell line derivation [6], [7]. For example, murine cells have much longer telomeres than human cells, which may allow for retention of telomerase activity [7]. It is possible that fish basal telomerase activity (at least in some species such as tilapia) is higher than in other species (e.g., humans), thus allowing for maintenance of telomere ends and successful immortalization of cells. An analysis of telomerase activity could therefore potentially provide insight into relative rates of success that can be expected for spontaneous immortalization of cells derived from different species.

### Rho kinase inhibitor/3T3 feeder layer supplementation induces immortalization of tilapia lip epithelial-like cells

In contrast to spontaneously immortalized brain cells (OmB), which transitioned a fibroblast phenotype upon immortalization, the lip cell line (OmL) retained an epithelial-like phenotype after ROCK/feeder layer-induced immortalization. The Rho family of proteins consists of small GTPases that are involved in many intracellular signaling processes including cell polarity, motility, proliferation, and apoptosis [14]. ROCK is a downstream effector protein of the GTP-bound form of Rho A [53]. Inhibition of this kinase was found to be an effective immortalization method for mammalian epithelial cells, such as human keratinocytes [9], [10]; however, this ROCK activity has not been reported in aquatic species. In this study, supplementation of medium with ROCK inhibitor in combination with the use of irradiated 3T3 feeder cells permitted immortalizing tilapia epithelial-like cells from lip tissue. This technique appears very promising for fish cells because epithelial-like cell characteristics can be selected for and preserved. Immortalization occurs more rapidly than without these additions and bypasses the period of crisis.

### Phenotypes of newly derived tilapia cell lines are distinct

In-depth characterization of all cellular properties for each cell line was beyond the scope of this study because our primary goal was to validate the physiological relevance of these cell lines as models for mechanistic studies of osmotic stress responses. Instead, a quantitative proteomic comparison was included in order to identify a few specific cell markers that could differentiate the two newly derived tilapia cell lines (OmB, OmL). Comparison of OmB and OmL proteomes yielded surprisingly distinct expression of protein biomarkers between the two, which supports the notion that these cell lines are comprised of different cell types. Specifically, OmB expressed higher abundances of proteins that have been documented in brain (astrocytic phosphoprotein PEA-15, hematological and neurological expressed 1 (HN1) protein, neurofilament heavy polypeptide, and optineurin). Astrocytic phosphoprotein PEA-15 is a protein kinase C substrate and is highly abundant in the brain of mammalian systems [54]. Interestingly, HN1 protein has been reported as a suppressor of differentiation in cells that are undergoing proliferation [55] and may have played an important role in the generation of a homogeneous cell population in OmB. Petzold et al. [56] identified neurofilament heavy polypeptide as one brain-specific marker for neurodegeneration. These protein targets support the notion that OmB cells have retained at least some of the cell properties from their tissue origin.

In contrast, OmL expressed much higher abundances of proteins that are characteristic of epidermis (keratin, integrin, tight junction, cingulin, junction plakoglobin, laminin). Keratins are the most abundant structural protein in the cytoplasm of epithelial cells (especially keratinocytes), and as a result, have commonly been used as an epithelial biomarker [57], [58]. Integrins and laminins are characteristic of epidermis and play a special role in structure integrity and stability [59], [60]. Specifically, laminins aid in providing attachment of the epidermis to the dermis and also enhance the formation of skin basement membranes [60], [61] Finally, tight junctions, cingulins, and junction plakoglobins are important structural components of epidermal cells [62]–[65]. Tight junctions of epidermis play an important role in barrier function, cell polarity, vesicle trafficking, differentiation, and proliferation [63].

Phenotypic classification of each cell line based on proteomics data was generally supported by cell morphology observations. Lip epithelium, like fin, has a very high regenerative ability, a property that likely contributed to the cell growth in culture, particularly in the early stages. The OmL cell line displayed an epithelial-like morphology (rounded cell shape), similar to keratinocytes. Interestingly, keratinocytes have been demonstrated to use osmolytes to maintain volume homeostasis [66]. Pathways associated with compatible osmolyte systems, specifically co-transporters, have been documented to be induced in keratinocytes under environmental stress [67], [68]. In contrast, the phenotypes of the TmB and OmB cell lines resemble endothelial (small rounded cell shape) and fibroblast-like (elongated, spindle cell shape) cells, respectively. It is possible that the use of a ROCK/3T3 feeder layer may be necessary to support the continuous growth of fish epithelial cells. In mammalian cultures, keratinocytes grown in the presence of serum depend on a fibroblast support layer to allow for successful proliferation in culture [69].

### Tilapia cell lines are highly osmotolerant

We found that the osmotolerance observed in the tilapia cells lines is higher than that documented for any other animal cell line. The observed high osmotic tolerance of euryhaline tilapia cells compared to mammalian cells is in agreement with the higher osmotolerance of tilapia *in vivo*. Cells from euryhaline fish species more frequently encounter osmotic stress in an aquatic environment compared to their terrestrial counterparts and thus may have evolved a higher tolerance to osmotic stress. Nevertheless, mammalian kidney cells, specifically renal inner medulla cells, are also regularly exposed to changes in osmolality due to the urinary concentrating mechanism [70], [71]. Many renal cell lines (e.g., MDCK, GRB-MAL1) are able to tolerate high osmolality in part due to the intracellular accumulation of organic osmolytes [72], [73]. However, the osmotolerance of mammalian renal cell lines (including inner medullary cells) is less than that of fish cells (generally not more than 600 mOsm/kg) when medium osmolality is acutely increased by adding sodium chloride [74]–[76].

It is possible that *myo*-inositol contained in the culture media may have an effect on the overall regulation of *myo*-inositol in the various cell lines via induction of other biochemical pathways. For example, *myo*-inositol in the media could potentially be fluxed into the cell via various osmolyte co-transporters (e.g., sodium/*myo*-inositol cotransporter, hydrogen/*myo*-inositol transporter) and may in turn also have an effect on the regulation of the MIB pathway enzymes. Extracellular *myo*-inositol and other osmolytes may play an important role in early stages of hyperosmotic acclimation. In the future, it would be interesting to determine if supplementation of media with *myo*-inositol has any effect on the osmotolerance of the tilapia cell lines. Interestingly, a study by Garcia de Castro and Tunnacliffe [77] found that trehalose supplementation increased osmotolerance in mammalian cells.

A recent study used a cichlid astroglial cell line and found that this cell line survived hypo-osmotic challenges and appeared to employ a compensatory regulatory volume decrease [41]. As expected, we found that the osmotolerance of OmL epithelial-like cells is significantly higher than that of OmB or TmB cells. In the whole organism, lip tissue is directly exposed to the external environment (water), unlike brain and heart tissues. One would predict cells of external tissues (e.g., lip) have a higher tolerance to hyperosmotic stress than cells of tissues that are entirely bathed in relatively homeostatic internal body fluids. Considering the maximum osmolality of 450 mOsm/kg that brain and endothelial cells are exposed to in intact fish [27], [78], it seems surprising that OmB and TmB cells are able to tolerate osmolalities as high as 700 mOsm/kg. However, disruption of osmotic homeostasis is known to cause major malfunction of the central nervous and cardiovascular systems, and the large margin of capacity in osmotolerance may speak to the critical importance of protecting vital organismal functions [79], [80]. The mechanisms for dealing with extreme osmotic challenges are well-developed in all three tilapia cell lines but they are significantly more effective in epithelial-like OmL cells. Interestingly, ion transporters, such as the sodium potassium ATPase pump proteins were highly expressed in the OmL protein dataset, but were not present in OmB. Thus, the main mechanisms that confer high osmotolerance to tilapia cells represent a constitutive property of the tilapia genome, which is most potently harnessed in epithelial cells.

### Tilapia cell lines respond to hyperosmotic stress by inducing MIB pathway enzymes

The MIB pathway consists of the enzymes MIPS and IMPA that convert glucose-6 phosphate to the compatible organic osmolyte, *myo*-inositol. This pathway has been identified as a major component of the response to hyperosmotic challenge in various tissues of tilapia [23], [24], [27], [28]. To assess the validity of TmB, OmB, and OmL cell lines as *in culture* models for studying the mechanisms underlying hyperosmotic MIB pathway induction, we characterized the mRNA expression of the MIB pathway enzymes, MIPS and IMPA. The MIB pathway is a highly conserved pathway in tilapia, across cell types and tissues (*in vivo*), for responding to hyperosmotic challenge. Specific MIPS splice variants and IMPA isoforms have been found to be highly induced at the mRNA and protein levels by hyperosmotic stress in a number of different tissues in intact tilapia [23], [24], [27], [28].

Overall, the previously described *in vivo* responses to hyperosmotic stress were reproduced in all tilapia cell lines. The same splice variants and isoforms of MIPS and IMPA were induced at the same or even more potency *in culture* compared to *in vivo*. It is interesting to note that cells at low (OmB: passage 11, OmL: passage 10) and high passages (TmB: passage 63) are able to utilize the MIPS and IMPA enzymes to respond to osmotic stress. This observation was also made across various putative cell types and reinforces the idea that the MIB pathway is a highly inducible and robust pathway during hyperosmotic stress. At the 450 mOsm/kg (physiologically relevant) exposure level, there is an earlier (at least 12 hr) maximum of the MIPS mRNA level compared to the 700 mOsm/kg (maximum osmotolerance) exposure level. It is possible that key cellular processes, such as gene expression, are inhibited during severe osmotic stress (700 mOsm/kg), which would explain why there is an observed delay in the transcriptional induction of the MIB pathway enzymes. In the early stages after hyperosmotic stress, cells are recovering from the mechanical stress of shrinkage and intracellular molecular crowding [70] by employing regulatory volume increase. During this time energy in the cell (generated via metabolizing glucose-6-phosphate in glycolytic pathways) is likely redirected to other immediate cellular processes, such as water regulation, cell shrinkage, and cytoskeletal reorganization [74]. Moreover, chromatin condensation has been documented in chondrocytes exposed to severe hyperosmolality [81]. Such condensation may physically inhibit gene expression until the cell recovers from the initial insult of the stress. The mechanisms discussed above collectively explain the delay in MIB pathway induction under severe (700 mOsm/kg) relative to moderate (450 mOsm/kg) hyperosmotic conditions.

The MIB enzyme transcriptional response in OmB and TmB was found to be more similar to each other than that of OmL. OmB and TmB showed a greater induction of IMPA relative to MIPS in both 450 mOsm/kg and 700 mOsm/kg conditions. Greater expression of IMPA1 relative to MIPS-160 is similar to what is reported *in vivo* [24], [27]. We found that the 700 mOsm/kg response of MIPS and IMPA mRNA abundances in the brain-derived OmB cell line was more similar to the *in vivo* response of brain tissue [27] than the 450 mOsm/kg, physiologically relevant treatment. However, the delay in MIB pathway induction *in culture* is likely a direct result of inhibition of critical cell functions by severe osmotic stress (see above). In contrast, the delay of MIB pathway induction *in vivo* results from a delay in plasma osmolality increase relative to increased environmental salinity. Brain cells in the whole organism only respond after the signal of increased environmental salinity is reflected in blood plasma osmolality and transduced across the blood-brain barrier to cerebrospinal fluid osmolality. In culture, interfaces such as the body surface or blood-brain barrier are not present and any osmotic change in cell culture medium is more rapidly reflected in responses of cell lines than in cells of intact fish.

Interestingly, in OmL we found that MIPS splice variant mRNA abundance was higher than that of IMPA isoforms in response to hyperosmotic challenge. It is still unclear why there is greater MIPS mRNA expression in this cell line compared OmB and TmB, but it is thought that conversion of G-6-P to inositol-3-phosphate by MIPS is the rate limiting step of the MIB pathway [82]. Interestingly, we also found that there is a greater induction in mRNA expression of the IMPA2 isoform in OmL compared to any other cell line. Additionally, proteomic comparative analyses between OmL and OmB revealed that an IMPA isoform was detected in OmL, but not in OmB. One possible explanation for this difference is that lip cells are exposed to the external environment (water) and more frequently encounter changes in extracellular osmolality. Also, osmolytes other than *myo*-inositol may contribute to osmotic homeostasis in epithelial cells of fish to deal with the more frequent osmolality changes. For example, taurine is known to be an important osmolyte in response to hyperosmotic stress in multiple tissues of tilapia [32]. Such coordinate regulation of multiple compatible organic osmolytes is a common physiological mechanism [25].

## Conclusions

In summary, we have illustrated that tilapia cell lines generated in this study are useful tools for studying how euryhaline fish respond to osmotic stress. We have clearly demonstrated that tilapia cell lines are highly osmotolerant and possess functional elements of the MIB pathway, which are robustly regulated in response to hyperosmotic challenge in all lines. Thus, this pathway is presumably a highly conserved and important aspect of response to osmotic challenge in tilapia cells. Intricate qualitative differences in the potency of MIB pathway induction and the regulation of MIPS and IMPA in the different cell lines renders the use of multiple tilapia cell lines highly valuable for dissecting mechanistic aspects of MIB pathway regulation during osmotic stress. For example, future work could determine whether knockdown (siRNA) or selective inhibition of MIB enzymes is capable of altering the phenotypic response of the cells (e.g., osmotolerance). Gene knockdown is rapidly expanding in fish [83], [84] and may be more feasible and economical in cell lines than in the whole organism. Overall, these newly established tilapia cell lines have an enormous potential for contributing to the fields of environmental physiology and ecotoxicology of fish.

## Supporting Information

File S1 **Quantitative protein data for expression of all significantly different proteins in OmB and OmL cells lines.** These data correspond to protein data shown on the PEAKS-generated heat map (Figure 2).
(XLSX)

File S2 **Quantitative peptide data for expression of all significantly different proteins in OmB and OmL cells lines.** These data are associated with protein data shown on the PEAKS-generated heat map (Figure 2).
(XLSX)

File S3 **Information for all proteins identified in OmB and OmL cell lines.** Protein data for each cell line are separated on different tabs.
(XLSX)

File S4 **Information for corresponding peptides of all proteins identified in OmB and OmL cell lines.** Peptide data for each cell line are separated on different tabs.
(XLSX)
